# Subclinical atherosclerosis burden predicts cardiovascular events in individuals with diabetes and chronic kidney disease

**DOI:** 10.1186/s12933-019-0897-y

**Published:** 2019-07-19

**Authors:** Ana Palanca, Esmeralda Castelblanco, Àngels Betriu, Hèctor Perpiñán, Berta Soldevila, José Manuel Valdivielso, Marcelino Bermúdez-Lopez, Carlos Puig-Jové, Manel Puig-Domingo, Per-Henrik Groop, Elvira Fernández, Núria Alonso, Didac Mauricio

**Affiliations:** 1 Department of Endocrinology & Nutrition, Health Sciences Research Institute & University Hospital Germans Trias i Pujol, Carretera Canyet S/N, 08916 Badalona, Spain; 2 Department of Medicine, Barcelona Autonomous University (UAB), Barcelona, Spain; 3 Department of Endocrinology & Nutrition, Hospital de la Santa Creu i Sant Pau & Institut d’Investigació Biomédica Sant Pau (IIB Sant Pau), Sant Quintí, 89, 08041 Barcelona, Spain; 4 Center for Biomedical Research on Diabetes and Associated Metabolic Diseases (CIBERDEM), Barcelona, Spain; 5 Vascular and Renal Translational Research Group, Institut de Recerca Biomèdica de Lleida, Lleida, Spain; 6 Biostatistics Unit, Institut de Recerca Biomèdica de Lleida, Lleida, Spain; 7 Folkhälsan Institute of Genetics, Folkhälsan Research Center, Helsinki, Finland; 8 Abdominal Center Nephrology, University of Helsinki and Helsinki University Central Hospital, Helsinki, Finland; 9 Research Program for Clinical and Molecular Metabolism, Faculty of Medicine, University of Helsinki, Helsinki, Finland; 10 Department of Diabetes, Central Clinical School, Monash University, Melbourne, VIC Australia

**Keywords:** Chronic kidney disease, Diabetes, Multiterritorial arterial ultrasound, Subclinical atherosclerosis, Cardiovascular events

## Abstract

**Background:**

Individuals with diabetes have remarkably high rates of cardiovascular morbidity and mortality. However, the incremental cardiovascular risk in diabetes is heterogeneous and has often been related to renal involvement. The purpose of this study was to analyse the prognostic value of subclinical atherosclerosis in determining the incidence of first cardiovascular events (CVEs) in individuals with diabetes and chronic kidney disease (CKD) compared to CKD individuals without diabetes.

**Methods:**

We included data from individuals with CKD with and without diabetes, free from pre-existing cardiovascular disease, from the NEFRONA cohort. Participants underwent baseline carotid and femoral ultrasound and were followed up for 4 years. All CVEs during follow-up were registered. Bivariate analysis and Fine–Gray competing risk models were used to perform the statistical analysis.

**Results:**

During the mean follow-up time of 48 months, a total of 203 CVE was registered. 107 CVE occurred among participants without diabetes (19.58 per 1000 person-years) and 96 CVE occurred among participants with diabetes (44.44 per 1000 person-years). Following the competing risk analysis, the variables predicting CVEs in CKD individuals without diabetes were the number of territories with plaque at baseline (HR 1.862, 95% CI [1.432;2.240]), age (HR 1.026, 95% CI [1.003;1.049]) and serum concentrations of 25-OH vitamin D (HR 0.963, 95% CI [0.933;0.094]). The only variable predicting CVEs among CKD participants with diabetes was the number of territories with plaque at baseline (HR 1.782, 95% CI [1.393, 2.278]). For both models, concordance (C) index yielded was over 0.7.

**Conclusions:**

The burden of subclinical atherosclerosis is the strongest predictor of future CVEs in diabetic individuals with CKD. Early detection of subclinical atherosclerotic burden by multiterritorial vascular ultrasound could improve CVE prediction in this population.

**Electronic supplementary material:**

The online version of this article (10.1186/s12933-019-0897-y) contains supplementary material, which is available to authorized users.

## Background

It is well established that individuals with diabetes have an increased risk of developing cardiovascular disease (CVD) as well as poorer cardiovascular outcomes compared to the general population [1]. For the last two decades, diabetes has been considered an equivalent condition to CVD [2], and this heightened cardiovascular risk has been emphasized in former clinical guidelines [3, 4]. That said, some studies have reported that although individuals with diabetes show a two to fourfold increased risk of CVD, this risk is not equivalent to that of individuals who have had a cardiovascular event (CVE) [5–8]. These data support the concept of heterogeneity in cardiovascular risk within the diabetic population and therefore underline the need for cardiovascular risk stratification [9, 10].

On the other hand, quantification of the burden of atherosclerotic plaque assessed by noninvasive ultrasonography is a strong predictor for CVEs and, hence, is considered a valid tool for cardiovascular risk stratification [11, 12]. In addition, several studies support the value of measuring subclinical atherosclerosis (SA) in multiple arterial territories for an improved CVE predictive value and, thus, a more accurate cardiovascular risk stratification [13, 14]. In addition, the substantial impact of declining renal function and albuminuria on cardiovascular risk has been broadly acknowledged in the literature [15]. In fact, it has been reported that in individuals with diabetes, the presence of chronic kidney disease (CKD) is associated with an increased risk of CVD, explaining in part their heightened burden of CVD [16].

To date, there is limited evidence describing the specific prevalence of SA in individuals with both diabetes and CKD. Recently, our group reported that SA is more prevalent, carries a higher plaque burden and is more rapidly progressive in individuals with CKD and diabetes [17]. In addition, our group also reported that renal individuals show a higher prevalence of plaques [18, 19] and a higher incidence of future CVEs across all stages of CKD compared with controls [20].

Therefore, we examined the prognostic value of multiterritorial vascular evaluation and the extent of SA in predicting the incidence of new CVEs in individuals with renal disease and diabetes, using data from the National Observatory of Atherosclerosis in Nephrology (NEFRONA) study [21]. We hypothesized that the additional atherosclerotic plaque burden that involves having both conditions would be a marker of an increased risk of CVEs and contribute to the prediction of the incidence of CVEs among CKD individuals with diabetes in our cohort.

Thus, the aim of our study was to analyse the incidence of CVEs and its association with baseline subclinical atherosclerotic status in CKD individuals with and without diabetes.

## Methods

### Design and study population

The present study included 1747 individuals with CKD without diabetes and 698 individuals with CKD with diabetes from the NEFRONA cohort. The design, objective and methods of the NEFRONA study have been published in detail previously [21]. Briefly, the NEFRONA study is a multicentre, prospective observational study. We aimed to evaluate the prevalence and evolution of subclinical atheromatosis in CKD individuals, as well as the contribution of vascular imaging for a more precise cardiovascular risk assessment.

Between October 2010 and June 2012, 2445 CKD individuals with and without diabetes who were free from previous cardiovascular disease and were 18 to 75 years of age were recruited from 81 Spanish hospitals and dialysis clinics [19]. Participants with known CVD or who had undergone any carotid artery intervention were excluded. Other exclusion criteria were pregnancy, life expectancy of less than 12 months, any active infection or previous organ transplantation. Participants underwent baseline carotid and femoral ultrasound examinations. During a 4-year follow-up period, all cardiovascular events, noncardiovascular deaths and kidney transplantations were registered.

The Ethical Committees of all involved Spanish nephrology centres approved this study with the final approval by the Ethics committee board of the Hospital Universitario Arnau de Vilanova (Lleida, Spain). The investigation has been conducted according to the principles expressed in the Declaration of Helsinki, and all the included participants provided written informed consents.

### Clinical data and laboratory examinations

Information on the participant’s medical history, cardiovascular risk factors and drug use was collected at baseline. Dyslipidaemia was therefore defined as a recorded clinical diagnosis or current use of lipid-lowering medication. A detailed history was taken. A physical examination including standard vital tests and anthropometric measures, such as height, weight and waist–hip ratio, was performed [21]. A routine fasting blood test was carried out within 3 months of the vascular ultrasound examination, and biochemical parameters were obtained. The estimated glomerular filtration rate was determined using the Modification of Diet in Renal Disease Study formula (MDRD-4). High-sensitive C reactive protein (hsCRP) plasma concentrations were measured with an immunoturbidimetric method (Roche/Hitachi modular analytics). Serum concentrations of 25-OH vitamin D were determined by an ELISA (IDS, UK) as described previously [20].

### Diagnosis of diabetes mellitus

The criteria used to make the diagnosis of diabetes and, thus, to include a individual in the diabetes group have been reported previously [17]. The criteria used were as follows: a previous diagnosis of diabetes recorded in the individual’s medical history, a fasting plasma glucose ≥ 126 mg/dl or HbA1c ≥ 6.5% determined by laboratory testing or a current prescription of any anti-diabetic drug.

### Carotid and femoral imaging

B-mode ultrasound and colour Doppler examinations of carotid and femoral sites were performed using the Vivid BT09 apparatus (GE Healthcare, Waukesha, WI) equipped with a 6–13 MHz broadband linear array probe previously explained [21]. The presence of atheromatous plaque was assessed in ten vascular territories: internal, bulb and common carotid, and common and superficial femoral arteries. Atheromatous plaque was defined as intima media thickness (cIMT) > 1.5 mm protruding into the lumen, according to the ASE Consensus Statement [22] and the Mannheim cIMT Consensus [23]. The ultrasound examination was performed in a blinded fashion by three itinerant teams belonging to the UDETMA (Unit for Detection and Treatment of Atherothrombotic Diseases, Hospital Universitari Arnau de Vilanova, Lleida, Spain) using semi-automatic EchoPAC Dimension software (GE Healthcare) according to a standardized protocol. To evaluate intraobserver plaque assessment reliability, a sample of 20 randomly chosen subjects was assessed 3 to 5 times on different days, obtaining a kappa coefficient of 1 and, therefore, indicating excellent intraobserver reliability [20].

### Follow-up period and cardiovascular events

Participants were followed-up for 4 years. During the follow-up period, data on fatal and nonfatal cardiovascular events were recorded by the referring physician as reported previously [20]. The CVE were defined according to the International Classification of Diseases, Ninth Revision, Clinical Modification [ICD9-CM], which includes unstable angina, myocardial infarction, transient ischaemic attack, cerebrovascular accident, congestive heart failure, arrhythmia, peripheral arterial disease or amputation due to peripheral arterial disease, and aortic aneurism [21]. Cardiovascular mortality causes included myocardial infarction, arrhythmia, congestive heart failure, stroke, abdominal aortic aneurism, mesenteric infarction, and sudden death. CVEs and death were accurately recorded. Noncardiovascular death and kidney transplants were also recorded during follow-up.

### Statistical analysis

Data for quantitative variables are presented as a median and interquartile range, and qualitative variables are presented as a number and percentage. Participant characteristics were compared between nondiabetic and diabetic participants using the Mann–Whitney U test and Pearson’s Chi-square test for non-normal quantitative and categorical variables, respectively.

The association between potential risk factors and CVEs was investigated using bivariate analyses (Cox proportional hazards model). Significant variables in bivariate analyses and potential confounding factors were used to develop appropriate multiple models for estimating CVE risk under competitive risk conditions.

The Fine–Gray competing risk regression model was used to estimate the contribution of baseline patient characteristics to the cumulative incidence of CVEs. Noncardiovascular death and kidney transplantation were both considered competing events. Different models were fitted for CKD participants without diabetes as well as for CKD participants with diabetes. The model in each case was selected using the Bayesian information criteria [24]. A concordance index (C-index) was used to measure the discriminative power of the Fine–Gray models at 24 and 48 months. A statistical significance level of 0.05 was used. The statistical analysis was carried out with R statistical software version 3.3.1.

## Results

### Baseline patient characteristics and study endpoints

The participants included 1747 CKD individuals without diabetes and 698 CKD individuals with diabetes. The baseline characteristics of the participants are described in Table 1. The median follow-up time was 48 months in both groups.

**Table 1:** Population baseline characteristics

	CKD without diabetes N = 1747	CKD with diabetes N = 698	*p*-value
Gender, male	1047 (59.9%)	461 (66.0%)	0.006
CKD stage			< 0.001
CKD-3	662 (37.9%)	288 (41.3%)	
CKD-4/5	551 (31.5%)	256 (36.7%)	
RTT	534 (30.6%)	154 (22.1%)	
Age [years]	59.0 [48.0;67.0]	65.0 [56.0;70.0]	< 0.001
Current smoker	967 (55.4%)	404 (57.9%)	0.275
Hypertension	1554 (89.0%)	673 (96.4%)	< 0.001
25-OH vitamin D [ng/ml]	15.4 [11.4;19.6]	13.8 [10.4;18.5]	< 0.001
eGFR [ml/min per 1.73 m^2^]	31.6 [20.9;44.3]	31.7 [21.5;44.2]	0.812
Glucose [mg/dl]	92.0 [85.0;101]	133 [108;164]	< 0.001
Total cholesterol [mg/dl]	177 [153;205]	171 [143;197]	< 0.001
HDL cholesterol [mg/dl]	48.0 [39.0;59.0]	44.0 [36.0;53.9]	< 0.001
LDL cholesterol [mg/dl]	103 [82.0;123]	92.0 [71.4;113]	< 0.001
non-HDL cholesterol [mg/dl]	128 [105;153]	122 [100;148]	0.011
Triglycerides [mg/dl]	118 [89.0;162]	147 [103;205]	< 0.001
hsCRP [mg/dl]	1.86 [0.90;4.17]	2.58 [1.16;5.84]	< 0.001
Antidiabetic treatment			< 0.001
Diet	–	166 (23.8%)	
Oral hypoglycemic drugs	–	164 (23.5%)	
Insulin treatment	–	368 (52.7%)	
HbA1c [g/dl]	5.40 [5.10;5.70]	6.80 [6.10;7.80]	< 0.001
Albumin/creatinine ratio mg/g	89.1 [11.2;384]	149 [18.6;601]	0.001
Pulse pressure [mmHg]	56.0 [47.0;69.0]	68.0 [54.0;81.0]	< 0.001
Antihypertensive treatment	1506 (86.2%)	650 (93.1%)	< 0.001
Lipid-lowering drugs	975 (55.8%)	465 (66.6%)	< 0.001
Number of plaques	1.00 [0.00;3.00]	3.00 [1.00;5.00]	< 0.001
Presence of any plaque	1138 (65.1%)	570 (81.7%)	< 0.001
Presence of carotid plaques	901 (51.6%)	502 (71.9%)	< 0.001
Carotid plaque [only]	291 (16.7%)	165 (23.6%)	< 0.001
Presence of femoral plaques	847 (48.5%)	405 (58.0%)	< 0.001
Femoral plaque [only]	237 (13.6%)	68 (9.74%)	0.012
> 2 territories with plaque	853 (48.8%)	465 (66.6%)	< 0.001
Carotid and femoral plaques	610 (34.9%)	337 (48.3%)	< 0.001
Follow-up time [months]	48.4 [27.1;52.0]	48.3 [25.7;52.0]	0.675

*CKD* chronic kidney disease, *RRT* renal replacement therapy, *eGFR* estimated glomerular filtration rate determined by the Modification of Diet in Renal Disease Study formula (MDRD-4), *HDL* high density lipoprotein, *LDL* low density lipoprotein, *hsCRP* high sensitivity C-reactive protein

During the mean follow-up time of 48 months, a total of 203 CVE was registered, of which 107 occurred among participants without diabetes and 96 among participants with diabetes. Corresponding CVE rates were 19.58 versus 44.44 per 1000 person-years, respectively (Additional file 1: Figure S1). We also observed that among diabetes participants, CVE rates increased with declining renal function: CVE rates were 30.90 per 1000 person-years in CKD-3 versus 41.14 per 1000 person-years in CKD-4/5 and 100.67 per 1000 person-years in individuals on renal replacement therapy (RRT) (*p*-*trend *< 0.001). This tendency was milder among renal participants without diabetes: CVE rates were 15.04 per 1000 person-years in CKD-3 versus 18.19 per 1000 person-years in CKD-4/5 versus 31.72 per 1000 person-years for RRT (*p*-*trend *< 0.001). (Additional file 1: Figure S1).

Among women without diabetes, the CVE rate during follow-up was 16.04 per 1000 person-years and in men without diabetes, it was 22.05 per 1000 person-years. Among women with diabetes, the CVE rate was 32.17 per 1000 person-years, whereas among men with diabetes, it was 50.92 per 1000 person-years. Women with diabetes presented with more CVEs than men without diabetes (32.17 versus 22.05 per 1000 person-years, respectively; *p *= 0.0019), and this was observed consistently across all stages of CKD. In line with this result, stage 3 CKD women with diabetes experienced a slightly higher rate of incident CVEs compared to men with diabetes and stage 3 CKD (31.52 versus 30.67 per 1000 person-years, respectively) (Additional file 1: Figure S1).

### Factors associated with CVE

Among CKD participants without diabetes, the bivariate analysis showed that age (HR 1.04, 95% CI [1.02;1.06]), RRT (HR 2.23, 95% CI [1.40;3.55]), current smoker (HR 1.60, 95% CI [1.08;2.39]), serum concentration of HDL-cholesterol (0.98, 95% CI [0.96;0.99]), serum concentration of hsCRP (HR 1.01, 95% CI [1.00;1.03]) and pulse pressure values (1.02, 95% CI [1.01;1.03]) were associated with the occurrence of CVEs during the follow-up period (Additional file 1: Table S1). Among CKD participants with diabetes, factors associated with a CVE were RRT (HR 3.58, 95% CI [2.16;5.93]) and insulin treatment (HR 1.87, 95% CI [1.06;3.30]). Additionally, in this group of CKD participants with diabetes, being a female was associated with fewer CVEs (0.63, 95% CI [0.40;1.00]) as well as having better renal function (HR 0.98, 95% CI [0.96;1.00]) (Additional file 1: Table S1). In both groups, it was found that serum concentrations of 25-OH vitamin D were inversely associated with the incidence of CVE, participants without diabetes HR 0.96, 95% CI [0.93;0.99], and participants with diabetes HR 0.95, 95% CI [0.92;0.98] (Additional file 1: Table S1).

In both the nondiabetic and diabetic study groups, the bivariate analysis showed that the presence of plaque (HR 5.83, 95% CI [2.95;11.54] and HR 3.39, 95% CI [1.48;7.75], respectively) and the number of territories with basal plaque (HR 1.26, 95% CI [1.18;1.35] and HR 1.18, 95% CI [1.09;1.27], respectively) as well as having more than two vascular territories affected with plaque at baseline (HR 3.26, 95% CI [2.09;5.07] and HR 3.35, 95% CI [1.87;6.01], respectively) were associated with suffering from a CVE during follow-up (Table 2). To the same extent, the presence of atherosclerotic plaques in both vascular sites, carotid and femoral, was also found to be positively associated with incident CVEs in both groups (HR 2.57, 95% CI [1.75;3.78] and HR 2.09, 95% CI [1.37;3.17], respectively) (Table 2).

**Table 2:** Bivariate unadjusted analysis of the plaque at baseline according to the incidence of cardiovascular events

	CKD without diabetes				CKD with diabetes			
	No CVE N = 1640	CVE N = 107	HR [95% CI]	*p* value*	No CVE N = 602	CVE N = 96	HR [95% CI]	*p* value*
Territories with plaque	1.0 [0.0;3.0]	3.0 [2.0;5.0]	1.26 [1.18;1.35]	< 0.001	2.0 [1.0;5.0]	4.0 [2.0;6.0]	1.18 [1.09;1.27]	< 0.001
Presence of plaque	1040 (63.4%)	98 (91.6%)	5.83 [2.95;11.54]	< 0.001	480 (79.7%)	90 (93.8%)	3.39 [1.48;7.75]	0.004
Presence of carotid plaque	815 (49.7%)	86 (80.4%)	3.80 [2.36;6.12]	< 0.001	418 (69.4%)	84 (87.5%)	2.86 [1.56;5.24]	0.001
Carotid plaque [only]	267 (16.3%)	24 (22.4%)	1.41 [0.90;2.22]	0.137	143 (23.8%)	22 (22.9%)	0.92 [0.57;1.49]	0.748
Presence of femoral plaque	773 (47.1%)	74 (69.2%)	2.42 [1.60;3.64]	< 0.001	337 (56.0%)	68 (70.8%)	1.81 [1.17;2.82]	0.008
Femoral plaque [only]	225 (13.7%)	12 (11.2%)	0.83 [0.45;1.51]	0.538	62 (10.3%)	6 (6.25%)	0.56 [0.25;1.29]	0.173
> 2 territories with plaque	772 (47.1%)	81 (75.7%)	3.26 [2.09;5.07]	< 0.001	382 (63.5%)	83 (86.5%)	3.35 [1.87;6.01]	< 0.001
Carotid and femoral plaque	548 (33.4%)	62 (57.9%)	2.57 [1.75;3.78]	< 0.001	275 (45.7%)	62 (64.6%)	2.09 [1.37;3.17]	0.001

*CVE* fatal and non-fatal cardiovascular event

*p values correspond to HR. Chi-squared test for trend in proportions p < 0.005

Following the competing risk model analysis, the variables predicting CVE in CKD participants without diabetes were the number of territories with plaque at baseline (HR 1.862, 95% CI [1.432;2.240]), age (HR 1.026, 95% CI [1.003;1.049]) and serum concentrations of 25-OH vitamin D (HR 0.963, 95% CI [0.933;0.094]) (Table 3). On the other hand, among CKD participants with diabetes, the only variable that predicted CVEs was the number of territories with plaque at baseline (HR 1.782, 95% CI [1.393;2.278]) (Table 3). Estimated C-index values over 0.7 were found for the selected model in each group (Table 3).

**Table 3:** Fine and Gray multiple regression to model incidence of cardiovascular events

	CKD without diabetes		CKD with diabetes	
	HR [95% CI]	*p*-value	HR [95% CI]	*p*-value
Age	1.026 [1.003;1.049]	0.024	–	–
Baseline PL [sq]	1.862 [1.432;2.420]	< 0.001	1.782 [1.393;2.278]	< 0.001
25-OH vitamin D	0.963 [0.933;0.994]	< 0.001	–	–
C-index (24 months)	78.88		71.82	
C-index (48 months)	75.63		71.49	

The variables introduced to build the model were: gender, age in years, smoking, CKD stage, HDL cholesterol, 25-OH vitamin D and the square root of number of territories with plaque(s) at baseline, and glycated haemoglobin, oral treatment, and insulin treatment only for DM patients

*CKD* chronic kidney disease, *Baseline PL [sq]* the square root of number of territories with plaque

## Discussion

In the present study, we found that in CKD participants with diabetes and without known CVD, an increased burden of basal atherosclerotic plaque translates into an increased risk of incident CVEs. We also demonstrated that the number of vascular territories affected with plaque is the strongest associated factor for future CVEs in CKD individuals with diabetes, whereas in nondiabetic CKD individuals, other factors influenced the occurrence of CVE, such as age and serum concentrations of 25-OH vitamin D. To the best of our knowledge, this is an original work showing that in individuals with CKD and diabetes, multiterritorial vascular ultrasonography could help in future CVE prediction.

### Atherosclerosis burden and CVE

Atherosclerosis is a diffuse condition. Historically, necropsy examinations have revealed extensive atherosclerotic lesions in subjects experiencing fatal CVEs [25], thus linking CVD and atherosclerosis burden. Data from the literature have shown that the detection of preclinical atherosclerosis improved cardiovascular risk prediction beyond traditional cardiovascular risk factors [26, 27]. In addition, the quantification of plaque burden improved risk prediction even further [27–29]. Proxy measures of atherosclerosis burden, including noninvasive techniques, such as coronary artery calcification (CAC) score and vascular ultrasonography, have been the object of multiple investigations as a tool to predict incident CVEs. In individuals without established CVD, previous studies have reported that atherosclerosis burden, measured either by CAC score or plaque burden at carotid or femoral sites, is a strong and independent predictor of CVEs [30, 31]. A high atherosclerotic burden has also been associated with increased cardiovascular and total mortality risk [32–34], even in very elderly people [35]. Additionally, data from prior studies including renal participant cohorts have also demonstrated the relationship between atherosclerosis burden and CVD. A study from our group published in 2017 [20] found that the number of arterial territories affected with atherosclerotic plaque predicted the occurrence of CVEs in a cohort of asymptomatic renal individuals, including all-stages of CKD. Likewise, in a work that followed 226 individuals on haemodialysis for 5 years with carotid ultrasonography [36], plaque number was found to be an independent marker of fatal CVEs.

Concerning individuals with diabetes, the atherosclerotic burden measured by the CAC score has been shown to predict CVEs in asymptomatic subjects [7], while the presence of carotid and/or lower limb atherosclerosis assessed by ultrasonography has been shown to be associated with the prevalence of CVD [37], with the risk being greater in those subjects with concomitant carotid and femoral atherosclerosis.

### Other factors associated with CVE

The analysis of associated factors with the incidence of CVEs in our cohort of individuals has shown that age and serum 25-OH vitamin D concentrations were predictive of CVEs only in CKD individuals without diabetes, while the number of territories with plaque at baseline was predictive of CVEs both in individuals with and without diabetes.

Age is a well-known factor for CVD [38]. In our study, age was independently associated with CVEs in CKD individuals without diabetes but not in those with diabetes, inferring that individuals with diabetes present with a higher incidence of CVEs at younger ages, as has been reported in the literature [39]. Likewise, the association between 25-OH vitamin D serum concentrations and the incidence of CVE found in the present study in individuals without diabetes is in keeping with studies that appear to link vitamin D deficiency to CVD [40].

In the current analysis, there were no other risk factors that improved the assessment of CVE risk once added to the number of territories with plaque. Strikingly, the only factor predicting CVEs in this group with diabetes was atherosclerotic burden.

In fact, in our study, sex was not a risk factor for the incidence of CVEs in individuals with or without diabetes. In the general population, it is known that nondiabetic women have fewer CVEs than nondiabetic men of the same age. However, this advantage appears to be lost in the presence of diabetes [41–45]. The present study is in agreement with these findings showing that the loss of the protective cardiovascular sex effect is also observed in women with diabetes and CKD. This statement is reinforced by the fact that the incidence of CVEs in women from our cohort with diabetes was higher than that in men without diabetes, as well as by the data that the incidence of CVEs was similar in women with diabetes in stage 3 CKD compared to males with diabetes with similar kidney function.

Regarding cardiovascular risk prediction in patients with diabetes, Colom et al. have recently described that in patients with type 1 diabetes without previous cardiovascular disease alterations in the composition of HDL are associated with subclinical coronary artery disease as well as with an increased volume of epicardial adipose tissue suggesting that HDL composition may be a link between coronary atherosclerosis and the accumulation of this type of adipose tissue in these subjects [46]. Also in subjects with type 1 diabetes free from CV disease, our group has recently described that presence of advanced stages of diabetic retinopathy are associated with presence and burden of subclinical carotid atherosclerosis [47]. On the other hand, in healthy normotensive and normoglycaemic subjects classical risk factors, such as age and blood pressure, have been found to be associated with subclinical vascular damage while no association has been found with plasma biomarkers involved in the inflammatory process of atherosclerosis [48].

Finally, as we have demonstrated in the present work, quantification of atherosclerotic lesions by vascular ultrasonography is a feasible and reliable tool for CVE prediction. It should also be noted that new three-dimensional [28] and contrast-enhanced ultrasonography [49] could improve vascular assessment even further and thus contribute to refining the early prediction of CVEs.

### Limitations

A limitation of the current work relates to the baseline clinical characteristics relevant to diabetes that were not available due to the inherent nature of the NEFRONA study that was initially designed to investigate renal disease and cardiovascular risk. In addition, the endpoints registered during the observation period entailed the discontinuation of the follow-up after a first CVE, after nonCV death or after renal transplantation. However, this has been considered and mitigated with the competing risk analysis.

## Conclusions

CVD screening in asymptomatic individuals with diabetes remains controversial [50]. Moreover, CVD risk stratification remains an important challenge in the CKD population. However, our results shed some light on this matter. We have demonstrated that multiterritorial ultrasonography is a valid and strong noninvasive tool to help predict CVEs among diabetic individuals with CKD. In this subgroup of high-risk individuals, quantifying SA by the combined application of carotid and lower extremity vascular ultrasonography could improve CVE prediction and may help identify those individuals with higher CV risk. The strategies implemented in routine practice to efficiently quantify subclinical arterial lesions in diabetic individuals with CKD might be of clinical benefit and should be considered with a special emphasis on younger women with diabetes.

## Additional file

**Additional file 1: Figure S1.** Cardiovascular event incidence rates per 1000 person-years according to chronic kidney disease stage, diabetes status and gender. **Table S1.** Bivariate analysis of baseline characteristics in the NEFRONA cohort by incidence of cardiovascular events.

## Data Availability

Primary material is held by the authors.

## Abbreviations

CKD: chronic kidney disease
CVEs: cardiovascular events
SA: subclinical atherosclerosis
CVD: cardiovascular disease
cIMT: carotid intima media thickness
RRT: renal replacement therapy
CAC: coronary artery calcification
MI: myocardial infarction
