# Supplementary material for: Subclinical atherosclerosis burden predicts cardiovascular events in individuals with diabetes and chronic kidney disease
Source: Cardiovasc Diabetol. 2019 Jul 19;18:93. doi: 10.1186/s12933-019-0897-y (PMC6639953; doi:10.1186/s12933-019-0897-y)
Supplement: Supplementary file 1 — Additional file 1: Figure S1. Cardiovascular event incidence rates per 1000 person-years according to chronic kidney disease stage, diabetes status and gender. Table S1. Bivariate analysis of baseline characteristics in the NEFRONA cohort by incidence of cardiovascular events. [file 12933_2019_897_MOESM1_ESM.docx]

**Additional file 1**

**Figure S1.** Cardiovascular event incidence rates per 1,000 person-years according to chronic kidney disease stage, diabetes status and gender.
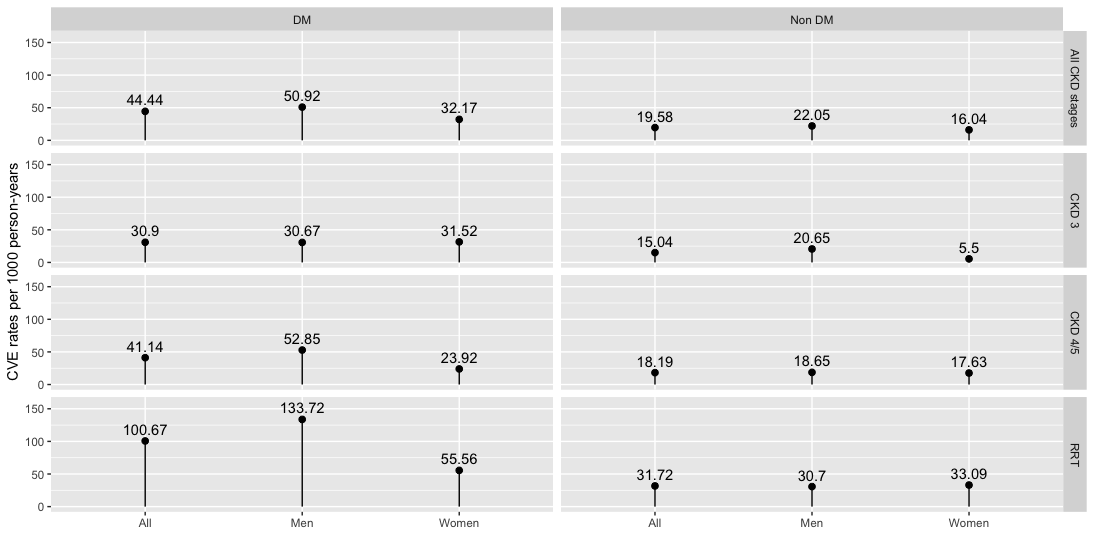
CVE: fatal and non-fatal cardiovascular event; CKD**:** chronic kidney disease; RRT**:** renal replacement therapy

**Table S1.** Bivariate analysis of baseline characteristics in the NEFRONA cohort by incidence of cardiovascular events

|  | **CKD without diabetes** | | | | **CKD with diabetes** | | | |
| --- | --- | --- | --- | --- | --- | --- | --- | --- |
|  | **No event N=1640** | **Event N=107** | **HR [95%CI]** | ***p* value** | **No CVE N=602** | **CVE N=96** | **HR [95%CI]** | ***P* value** |
| Gender: |  |  |  |  |  |  |  |  |
| Male | 976 (59.5%) | 71 (66.4%) | Ref. | Ref. | 389 (64.6%) | 72 (75.0%) | Ref. | Ref. |
| Female | 664 (40.5%) | 36 (33.6%) | 0.72 [0.48;1.08] | 0.115 | 213 (35.4%) | 24 (25.0%) | 0.63 [0.40;1.00] | 0.050 |
| CKD stage: |  |  |  |  |  |  |  |  |
| CKD-3 | 625 (38.1%) | 37 (34.6%) | Ref. | Ref. | 256 (42.5%) | 32 (33.3%) | Ref. | Ref. |
| CKD-4/5 | 517 (31.5%) | 34 (31.8%) | 1.22 [0.76;1.94] | 0.412 | 222 (36.9%) | 34 (35.4%) | 1.35 [0.83;2.19] | 0.220 |
| RTT | 498 (30.4%) | 36 (33.6%) | 2.23 [1.40;3.55] | 0.001 | 124 (20.6%) | 30 (31.2%) | 3.58 [2.16;5.93] | <0.001 |
| Age [years] | 59.0 [47.0;67.0] | 65.0 [58.5;70.0] | 1.04 [1.02;1.06] | <0.001 | 64.5 [56.0;70.0] | 65.0 [58.0;70.0] | 1.01 [0.99;1.03] | 0.414 |
| Current smoker | 897 (54.7%) | 70 (65.4%) | 1.60 [1.08;2.39] | 0.020 | 342 (56.8%) | 62 (64.6%) | 1.36 [0.90;2.07] | 0.148 |
| Arterial hypertension | 1455 (88.7%) | 99 (92.5%) | 1.47 [0.71;3.01] | 0.297 | 579 (96.2%) | 94 (97.9%) | 1.43 [0.35;5.80] | 0.617 |
| 25-OH Vitamin D [ng/ml] | 15.5 [11.6;19.7] | 13.9 [10.3;18.0] | 0.96 [0.93;0.99] | 0.007 | 14.0 [10.6;18.9] | 12.5 [9.19;16.7] | 0.95 [0.92;0.98] | 0.006 |
| eGFR [mL/min per 1.73 m2] | 31.7 [20.8;44.4] | 30.9 [21.1;43.2] | 0.99 [0.97;1.01] | 0.302 | 32.2 [22.1;44.5] | 28.0 [18.5;41.0] | 0.98 [0.96;1.00] | 0.038 |
| Glucose [mg/dl] | 92.0 [85.0;101] | 96.0 [86.0;105] | 1.01 [0.99;1.02] | 0.314 | 132 [108;161] | 147 [108;170] | 1.00 [1.00;1.00] | 0.346 |
| Total cholesterol [mg/dl] | 178 [154;205] | 174 [145;210] | 1.00 [0.99;1.00] | 0.752 | 172 [144;197] | 166 [139;198] | 1.00 [0.99;1.01] | 0.952 |
| HDL cholesterol [mg/dl] | 48.0 [39.0;59.0] | 45.0 [39.0;53.0] | 0.98 [0.96;0.99] | 0.002 | 44.0 [37.0;53.9] | 43.0 [32.0;52.0] | 0.99 [0.98;1.01] | 0.291 |
| LDL cholesterol [mg/dl] | 103 [82.0;123] | 101 [73.0;126] | 1.00 [0.99;1.00] | 0.437 | 92.5 [72.0;112] | 92.0 [71.0;118] | 1.00 [1.00;1.01] | 0.154 |
| non-HDL cholesterol [mg/dl] | 128 [105;152] | 132 [103;158] | 1.00 [1.00;1.01] | 0.555 | 122 [100;147] | 126 [96.0;155] | 1.00 [1.00;1.01] | 0.579 |
| Triglycerides [mg/dl] | 118 [88.0;162] | 134 [107;175] | 1.00 [1.00;1.00] | 0.060 | 147 [104;206] | 148 [91.8;192] | 1.00 [1.00;1.00] | 0.492 |
| hsCRP [mg/dl] | 1.82 [0.90;4.00] | 2.52 [1.24;7.32] | 1.01 [1.00;1.03] | 0.010 | 2.48 [1.12;5.44] | 3.10 [1.44;6.84] | 1.01 [0.99;1.04] | 0.248 |
| HbA1c [g/dl] | 5.40 [5.05;5.70] | 5.35 [5.10;5.73] | 0.71 [0.38;1.35] | 0.299 | 6.80 [6.10;7.80] | 6.90 [6.30;8.10] | 1.12 [0.96;1.31] | 0.143 |
| Antidiabetic treatment |  |  |  |  |  |  |  |  |
| Diet |  |  |  |  | 151 (25.1%) | 15 (15.6%) | Ref. | Ref. |
| Oral hypoglycemic drugs |  |  |  |  | 144 (23.9%) | 20 (20.8%) | 1.17 [0.60;2.28] | 0.649 |
| Insulin treatment |  |  |  |  | 307 (51.0%) | 61 (63.5%) | 1.87 [1.06;3.30] | 0.029 |
| Albumin/creatinine ratio mg/g | 86.2 [10.5;381] | 177 [22.4;451] | 1.00 [1.00;1.00] | 0.179 | 139 [18.0;565] | 279 [29.3;1309] | 1.00 [1.00;1.00] | 0.045 |
| Pulse pressure [mmHg] | 56.0 [47.0;68.0] | 61.0 [48.5;75.0] | 1.02 [1.01;1.03] | 0.002 | 66.5 [54.0;80.0] | 72.0 [55.8;83.2] | 1.01 [1.00;1.02] | 0.155 |

### CVE: fatal and non-fatal cardiovascular event; CKD: chronic kidney disease; RRT: renal replacement therapy; eGFR: estimated glomerular filtration rate determined by the Modification of Diet in Renal Disease Study formula (MDRD-4); HDL: high density lipoprotein; LDL: low density lipoprotein; hsCRP: [high sensitivity C-reactive](http://scholar.google.es/scholar?q=high+sensitivity+c+reactive&hl=en&as_sdt=0&as_vis=1&oi=scholart) protein.
